# Neuraminidase inhibitor susceptibility and neuraminidase enzyme kinetics of human influenza A and B viruses circulating in Thailand in 2010–2015

**DOI:** 10.1371/journal.pone.0190877

**Published:** 2018-01-11

**Authors:** Nipaporn Tewawong, Bindumadhav M. Marathe, Yong Poovorawan, Sompong Vongpunsawad, Richard J. Webby, Elena A. Govorkova

**Affiliations:** 1 Center of Excellence in Clinical Virology, Department of Pediatrics, Faculty of Medicine, Chulalongkorn University, Bangkok, Thailand; 2 Department of Infectious Diseases, St. Jude Children’s Research Hospital, Memphis, Tennessee, United States of America; University of Georgia, UNITED STATES

## Abstract

Amino acid substitutions within or near the active site of the viral neuraminidase (NA) may affect influenza virus fitness. In influenza A(H3N2) and B viruses circulating in Thailand between 2010 and 2015, we identified several NA substitutions that were previously reported to be associated with reduced inhibition by NA inhibitors (NAIs). To study the effect of these substitutions on the enzymatic properties of NA and on virus characteristics, we generated recombinant influenza viruses possessing either a wild type (WT) NA or an NA with a single I222V, S331G, or S331R substitution [in influenza A(H3N2) viruses] or a single D342S, A395T, A395V, or A395D NA substitution (in influenza B viruses). We generated recombinant (7:1) influenza A and B viruses on the genetic background of A/Puerto Rico/8/1934 (A/PR/8, H1N1) or B/Yamanashi/166/1998 (B/YAM) viruses, respectively. In contrast to the expected phenotypes, all the recombinant influenza A(H3N2) and B viruses carrying putative NA resistance substitutions were susceptible to NAIs. The *K*_m_ and *V*_max_ for the NAs of A/PR8-S331G and A/PR8-S331R viruses were higher than for the NA of WT virus, and the corresponding values for the B/YAM-D342S virus were lower than for the NA of WT virus. Although there was initial variation in the kinetics of influenza A and B viruses’ replication in MDCK cells, their titers were comparable to each other and to WT viruses at later time points. All introduced substitutions were stable except for B/YAM-D342S and B/YAM-A395V which reverted to WT sequences after three passages. Our data suggest that inferring susceptibility to NAIs based on sequence information alone should be cautioned. The impact of NA substitution on NAI resistance, viral growth, and enzymatic properties is viral context dependent and should be empirically determined.

## Introduction

Influenza A and B viruses are important human respiratory pathogens that cause annual epidemics worldwide. Anti-influenza prophylaxis and treatment with antiviral drugs are used to prevent and control influenza virus infections, particularly when vaccines are ineffective or unavailable [1]. The Centers for Diseases Control and Prevention (CDC) has reported high levels of resistance (>99%) to adamantanes among circulating seasonal influenza A(H3N2) and pandemic A(H1N1)pdm09 viruses [2]. Therefore, existing options for the prophylaxis and treatment of influenza virus infection are limited to a single class of antiviral drug, neuraminidase inhibitors (NAIs). Three NAIs, namely oral oseltamivir, inhaled zanamivir, and intravenous peramivir, are approved by the United States Food and Drug Administration and are recommended for the prophylaxis and treatment of patients infected with influenza A or B viruses [2].

The influenza neuraminidase (NA) is a tetrameric type II integral membrane protein with sialidase activity that is responsible for releasing newly produced viral particles from virus-infected cells [3, 4]. Based on their sequences, the NA genes of influenza viruses are classified as group 1 (N1, N4, N5, and N8), group 2 (N2, N3, N6, N7, N9) and influenza B NA [5]. In influenza A and B viruses, the NA active site is formed by highly conserved framework (E119, R156, W178, S179, D/N198, I222, E227, H274, E277, N294, and E425 [in N2 numbering]) and catalytic (R118, D151, R152, R224, E276, R292, R371, and Y406) residues [6–8]. NA amino acid substitutions located within or in close proximity to the NA active site can result in reduced inhibition of influenza viruses by NAIs and may affect the NA enzymatic properties, replication efficiency, transmissibility, and fitness of the virus *in vitro* and *in vivo* [9–12]. The various NA-subtype specific substitutions arise as a result of selection by different NAIs, and can be detected with different frequencies in clinical samples or in surveillance studies [12]. Although substitutions have been reported at novel residues in NAs, the effect of these substitutions on the NAI susceptibility of viruses with different genetic backgrounds is largely undetermined. Substitutions at residue 222 (T/V/M/L) conferred reduced susceptibility to NAIs and were found in seasonal A(H1N1), A(H1N1)pdm09, and highly pathogenic influenza A(H5N1) viruses. I222T/V NA substitutions were reported to cause reduced susceptibility of A(H5N1) viruses to NAIs, with 60- to 105-fold higher IC_50_s to oseltamivir when compared to the wild-type NAs [13]. Antiviral surveillance studies identified novel NA substitutions (S331R in influenza A(H3N2) viruses; D342S and A395E in influenza B viruses) that were associated with reduced inhibition by oseltamivir and peramivir (as indicated by higher IC_50_ values for these compounds) [14–17]. These substitutions were located outside the NA active site [6, 15]. A recent study demonstrated that an influenza A(H1N1)pdm09 variant containing double NA substitution at residues I427T/Q313R outside the NA active site had decreased NAI susceptibility, with altered NA properties and viral fitness [18].

Previously, we reported substitutions at residues 222 and 331 in the NA of influenza A(H3N2) viruses and at residues 342 and 395 in that of influenza B viruses circulating in Thailand [19]. However, the effect of these NA substitutions on the susceptibility to NAIs, the enzymatic properties, and the fitness of the viruses were not determined. Here, we applied reverse genetics and generated recombinant influenza A and B viruses possessing either wild-type (WT) NA or an NA with a single I222V, S331G, or S331R substitution [in influenza A(H3N2) viruses] or a single S342S, A395T, A395V, or A395S substitution [in influenza B viruses].We then used these recombinant viruses to study the properties (activity, enzyme kinetic, thermostability) of their NAs, their susceptibility to NAIs, their replication kinetics, and the genetic stability of the NA substitutions during passages in MDCK cells.

## Materials and methods

### Clinical samples, influenza viruses, and cells

The novel NA substitutions were identified in influenza A(H3N2) and B viruses in polymerase chain reaction (PCR)-positive respiratory samples from patients in Thailand (**Table 1**) [19]. Substitution-encoding NA gene sequences were amplified from these samples, which were obtained from the Center of Excellence in Clinical Virology at Chulalongkorn University in Thailand as part of the influenza surveillance program. The study protocol was approved by the Institutional Review Board (IRB) of the Faculty of Medicine at Chulalongkorn University (IRB No. 581/58). The study was conducted according to the ethical principles regarding human experimentation set out in the Declaration of Helsinki, and the IRB waived the need for consent because the samples were de-identified and anonymous.

**Table 1:** Novel NA substitutions in influenza A(H3N2) and B PCR-positive samples from Thailand used in the study.

Influenza virus	Subtype/lineage	Substitution in NA^a^	Recombinant influenza virus^b^	Accession no.^c^
A/Thailand/CU-B11870/2015	H3N2	Wild-type	A/PR8-WT	KX151204
A/Thailand/CU-B7418/2013	H3N2	I222V	A/PR8-I222V	KP336079
A/Thailand/CU-B11518/2015	H3N2	S331G	A/PR8-S331G	KX151190
A/Thailand/CU-B12139/2015	H3N2	S331R	A/PR8-S331R	KX151213
B/Thailand/CU-B11776/2015	B/Yam	D342S	B/YAM-D342S	KX151370
B/Thailand/CU-B5910/2011	B/Vic	A395T	B/YAM-A395T	JX513088
B/Thailand/CU-H3002/2011	B/Vic	A395D	B/YAM-A395D	JX513200
B/Thailand/CU-B10236/2014	B/Vic	A395V	B/YAM-A395V	KX151337

^a^ Numbering is based on an alignment of NAs from the following reference strains: A/Perth/16/2009 (H3N2) and B/Yamanashi/166/1998.

^b^ Designations of recombinant influenza viruses used in the study.

^c^ GenBank database accession numbers for the NAs listed.

Influenza B/Brisbane/60/2008 (B/BR/60/08) virus was obtained from the repository at St. Jude Children’s Research Hospital. Madin-Darby canine kidney (MDCK) cells (ATCC, Manassas, VA) were maintained in Eagle’s Minimum Essential Medium supplemented with 5% fetal bovine serum and a mixture of antibiotics and antimycotics (100 U of penicillin, 0.1 mg of streptomycin, and 0.25 μg of amphotericin B/mL). Human embryonic kidney 293T cells (ATCC, Manassas, VA) were maintained in Opti-MEM reduced-serum medium with GlutaMAX supplement at a final concentration of 2 mM (Life Technologies, Gaithersburg, MD) and no antibiotics.

### Cloning of NA into pHW2000 and pAD3000 plasmids

Influenza viral RNA was extracted using a RNeasy Kit (Qiagen, CA). The full-length NA genes (numbering is based on an alignment of NAs from the following reference strains: A/Perth/16/2009 (H3N2) and B/Yamanashi/166/1998 [20]) of influenza A(H3N2) and B viruses were amplified by one-step RT-PCR with primers previously described for the NA segments of influenza A [21] and B [22] viruses. PCR products were gel-purified (QIAquick Gel Extraction Kit, Qiagen) and then digested with *Bsa*I [21] or *Bsm*BI [22] restriction enzymes (New England Biolabs, Boston, MA). The plasmid vectors were digested with *Bsm*BI, and the NA segments were ligated with T4 DNA ligase (New England Biolabs, Boston, MA) into pHW2000 (for influenza A NAs) or pAD3000 (for influenza B NAs).

Plasmids were transformed into One Shot chemically competent Escherichia coli cells (Invitrogen, Carlsbad, CA), and positive clones were inoculated into Terrific Broth medium containing ampicillin. The positive clones were purified using HiSpeed Plasmid Maxi Kits (Qiagen, CA), and the plasmid DNA concentrations were determined by both UV spectrophotometry at 260 nm and analysis on agarose gels. The constructed plasmids were sequenced to ensure their identity with the field strains.

### Generation of recombinant influenza A and B viruses

Recombinant (7:1) influenza viruses carrying NAs of influenza A(H3N2) or B viruses circulating in Thailand (Table 1) were generated on a homogenous genetic background of A/Puerto Rico/8/1934 (H1N1) (A/PR8) and B/Yamanashi/166/1998 (B/YAM) influenza viruses, respectively. The A/PR8-WT was a 7:1 reassortant with the N2 NA of A/Thailand/CU-B11870/2015 in a background of A/PR8 (Table 1). The eight plasmids were transfected into 293T cells as previously described [22, 23]. The NA gene segment was amplified from the extracted viral RNA of rescued viruses by using one-step RT-PCR. The NAs of recombinant viruses were sequenced by the Hartwell Center for Bioinformatics and Biotechnology at St. Jude Children’s Research Hospital, using BigDye Terminator (version 3) chemistry and synthetic oligonucleotides. Samples were analyzed on Applied Biosystems 3700 DNA analyzers. The NA sequences were analyzed with SeqMan software (DNAStar, Madison, WI). Recombinant viruses were grown in the allantoic cavity of 10-day-old embryonated chicken eggs (Marshall Durbin, Birmingham, AL) for 72 h at 35°C (for influenza A viruses) or 33°C (for influenza B viruses). The virus-containing allantoic fluid was stored at −80°C until use. The 50% tissue culture infectious dose (TCID_50_) of each recombinant influenza virus was determined in MDCK cells incubated for 72 h at 35°C (for influenza A viruses) or 33°C (for influenza B viruses). The TCID_50_ values were calculated by the Reed-Muench method [24]. The HA titers were determined by using packed chicken red blood cells diluted to a hematocrit of 0.5%.

### NAI susceptibility

The NAIs (oseltamivir carboxylate, zanamivir, and peramivir) were dissolved in sterile distilled water, and aliquots were stored at −20°C until use. A fluorescence-based NA inhibition assay with the fluorogenic substrate 2′-(4-methylumbelliferyl)-α-D-N-acetylneuraminic acid (MUNANA; Sigma-Aldrich, St Louis, MO) was used to determine the 50% inhibitory concentration (IC_50_) values [25, 26]. Briefly, viruses were standardized to equivalent NA enzyme activity in the linear range of the curve and were mixed with various concentrations of NAI (ranged from 0.05 to 5000 nM in the final reaction mixture) in 96-well flat-bottom black opaque plates (Corning Costar, NY). The virus-NAI mixture was incubated at 37°C for 30 min prior to the addition of MUNANA and then incubated with substrate (100 μM final concentration) at 37°C for 30 min. The reaction was terminated by the addition of the stop solution (0.1 M glycine in 25% ethanol, pH 10.7). Fluorometric determinations were quantified with a Synergy 2 multi-mode microplate reader (BioTek Instruments, Winooski, VT) based on the release of the fluorescent product 4-methylumbelliferone (4-MU) using excitation and emission wavelengths of 360 and 460 nm, respectively. The IC_50_s of viruses were determined using the curve-fitting model in GraphPad Prism 5 software (GraphPad Software, La Jolla, CA). The phenotypic NAI susceptibility of influenza A and B viruses was evaluated based on the fold change in their IC_50_ values when compared to those of susceptible viruses from the same NA subtype/lineage, as previously described by the WHO Influenza Antiviral Working Group [14]. The inhibition was categorized as “normal” (a ≤ 10-fold change for influenza A and a ≤ 5-fold change for influenza B viruses); “reduced” (a 10–100-fold change for influenza A and a 5–50-fold change for influenza B viruses); or “highly reduced” (a ≥ 100-fold change for influenza A and a ≥ 50-fold change for influenza B viruses).

### NA enzyme activity and thermostability

The NA activity of recombinant viruses was measured by a fluorometric assay with MUNANA substrate as described above [25, 26]. The NA activity was normalized against the amount of infectious virus and presented as the quantity of 4-MU (in μg) formed per 1 log_10_TCID_50_ of the virus. The thermostability of the NA was determined by incubating viruses in a gradient thermocycler at 33, 37, 45, or 55°C for 15 or 30 min, then all viruses were brought to room temperature, and NA activity was subsequently measured [25, 26]. The NA activity was determined and expressed as a percentage of the NA activity of the virus at 4°C (unheated). The NA activity of each virus was calculated based on the mean of triplicate assays.

### NA enzyme kinetics assay

The enzyme kinetics (*V*_max_ and *K*_m_) of the NAs of recombinant influenza A and B viruses with a single NA amino acid substitution were determined as previously described [27]. First, the optimum dilution of recombinant virus to assess NA kinetic properties was determined. Briefly, 2-fold virus dilutions were incubated with the MUNANA substrate (100 μM), and NA activity was measured every 60 s for 60 min to identify virus dilution within a linear range of the curve that converts ≤ 15% of the total amount of MUNANA substrate and signal to noise ration > 10 across most of time course. We then measured the NA enzyme kinetics at pH 6.5 with enzyme buffer and a final concentration of MUNANA substrate ranging from 0.98 to 1000 μM. The reaction was monitored at 37°C in a total volume of 150 μL, and the fluorescence signal was detected every 60 s for 60 min using excitation and emission wavelengths of 360 and 460 nm, respectively. The MUNANA substrate affinity (*K*_m_) and velocity of catalysis (*V*_max_) values were calculated by fitting appropriate Michaelis-Menten equations to the data by using nonlinear regression in GraphPad Prism 5 software.

### Replication kinetics

Multi-step growth curves were determined for each recombinant influenza virus in MDCK cells. Confluent cell monolayers were infected with recombinant virus at a multiplicity of infection (MOI) of 1 or 0.001 TCID_50_/cell. The supernatant was collected at 6, 12, and 24 h (for an MOI of 1 TCID_50_/cell) and at 24, 48, and 72 h (for an MOI of 0.001 TCID_50_/cell) after virus inoculation and stored at −70°C until use. Virus titers were determined by TCID_50_ assays in MDCK cells.

### Statistical analysis

The NA activity, IC_50_ values, enzyme kinetic parameters (*K*_m_ and *V*_max_), and virus titers were compared by two-tailed Student’s *t*-tests. The results were considered significantly different if the *P*-value was less than 0.05.

## Results

### NAI susceptibility profiles

We used NA inhibition assays to determine the NAI susceptibility profiles of 7:1 recombinant influenza A and B viruses carrying single NA amino acid substitutions (**Table 2**). When compared to the IC_50_ value of the A/PR8-WT virus, the fold changes in the IC_50_ values of recombinant influenza A viruses were > 10-fold. These results demonstrated that A/PR8 viruses carrying NA from A(H3N2) virus with single I222V, S331R, or S331G NA substitutions were susceptible to NAIs. For the recombinant B/YAM viruses, the fold changes in the IC_50_ values were > 5-fold when compared to the IC_50_ of the B/BR/60/08 virus. These results revealed that B/YAM viruses carrying NAs from currently circulating influenza B viruses with single D342S, A395T, A395V, or A395D NA substitutions were susceptible to NAIs.

**Table 2:** NAI susceptibility profiles for recombinant influenza A and B viruses carrying a single NA substitution.

Recombinant influenza virus	NA enzyme inhibition assay (mean IC_50_ ± SD, nM)^a^		
	Oseltamivir carboxylate	Zanamivir	Peramivir
*Influenza A viruses*			
A/PR8-WT	0.3 ± 0.0 (1)	0.4 ± 0.1 (1)	0.1 ± 0.02 (1)
A/PR8-I222V	0.3 ± 0.1 (1)	0.5 ± 0.1 (1.3)	0.1 ± 0.01 (1)
A/PR8-S331G	0.5 ± 0.5 (1.6)	0.4 ± 0.1 (1)	0.3 ± 0.3 (3)
A/PR8-S331R	0.1 ± 0.1 (0.3)	0.4 ± 0.1 (1)	0.2 ± 0.06 (2)
*Influenza B viruses*			
B/BR/60/08	16.1 ± 7.8 (1)	3.0 ± 0.6 (1)	0.5 ± 0.33 (1)
B/YAM- D342S	8.3 ± 4.2 (0.5)	0.9 ± 0.3 (0.3)	0.4 ± 0.12 (0.8)
B/YAM-A395T	17.2 ± 4.5 (1.1)	3.4 ± 0.5 (1.1)	0.6 ± 0.08 (1.2)
B/YAM-A395D	12.3 ± 2.7 (0.8)	2.5 ± 0.5 (0.8)	0.5 ± 0.36 (1)
B/YAM-A395V	18.3 ± 7.2 (1.1)	1.2 ± 0.3 (0.4)	0.8 ± 0.12 (1.6)

^a^ The IC_50_ value is the concentration of NAI that inhibits viral NA activity by 50%. The values were obtained from three independent experiments. Data in parentheses represent the fold increase over the corresponding value for the A/PR8-WT or B/BR/60/08 viruses.

### NA enzyme activity

We investigated the effect of NA amino acid substitutions on the NA activity of 7:1 recombinant influenza A and B viruses by using a modified fluorescence-based assay. The NA enzyme sialidase activity was standardized according to the amount of infectious virus (per 1 log_10_TCID_50_) and expressed as the quantity of 4-MU (in μg) (**Table 3**). The NA substitutions at residue 331 (S331G and S331R) significantly decreased the NA activity of recombinant A/PR8 viruses as compared to that of A/PR8-WT NA (*P *< 0.001 and *P *< 0.01, respectively), whereas the I222V NA substitution slightly increased the NA activity (**Table 3**). In contrast, the B/YAM-D342S and B/YAM-A395T viruses had significantly higher NA activity when compared to the reference B/BR/60/08 virus (*P *< 0.01). The A395D substitution decreased the NA activity of recombinant B/YAM virus, whereas the A395V NA substitution increased the NA activity relative to that of the B/BR/60/08 virus (**Table 3**). Thus, the S331G substitution had the greatest impact on the NA activity of recombinant A/PR8 viruses, followed by the S331R substitution, and the D342S and A395T substitutions had the greatest effect on the NA activity of recombinant B/YAM viruses.

**Table 3:** NA activity and NA enzyme kinetics of recombinant influenza A and B viruses.

Recombinant influenza virus	Virus yield, MDCK cells (log_10_TCID_50_/mL)	NA enzymatic properties			
		NA activity^a^	*K*_m_ (μM)^b^	*V*_max_ (μM/min)^b^	*V*_max_ ratio^c^
*Influenza A viruses*					
A/PR8-WT	9.0 ± 0.5	41.6 ± 0.7	55.3 ± 3.3	0.08 ± 0.00	1.0
A/PR8-I222V	9.3 ± 0.1	45.8 ± 0.2	59.2 ± 0.8	0.09 ± 0.01	1.1
A/PR8-S331G	8.3 ± 0.4	4.3 ± 0.6***	71.7 ± 1.1**	0.14 ± 0.00***	1.8
A/PR8-S331R	9.4 ± 0.1	31.9 ± 0.9**	80.5 ± 5.2**	0.11 ± 0.01**	1.4
*Influenza B viruses*					
B/BR/60/08	5.6 ± 0.1	30.2 ± 3.0	20.3 ± 1.1	0.09 ± 0.01	1.0
B/YAM- D342S	7.5 ± 0.0	50.3 ± 0.9**	13.3 ± 0.1**	0.07 ± 0.00*	0.8
B/YAM-A395T	7.5 ± 0.0	58.2 ± 0.5**	17.8 ± 0.4*	0.08 ± 0.00*	0.9
B/YAM-A395D	7.5 ± 0.0	24.6 ± 0.9	31.5 ± 6.9	0.11 ± 0.01	1.2
B/YAM-A395V	7.5 ± 0.0	42.1 ± 5.3	18.1 ± 0.1	0.10 ± 0.00	1.1

^a^ Values are the mean ± SD from three independent experiments; they are expressed as the quantity of 4-MU (in μg) per 1 log_10_TCID_50_ of the virus.

^b^ Values are the mean *K*_m_ and *V*_max_ from three independent experiments ± SD.

^c^ Ratio of the *V*_max_ of the NA of the respective recombinant viruses to the *V*_max_ of the WT NA.

*P < 0.05

**P < 0.01; and

***P < 0.001 as compared to A/PR8-WT or B/BR/60/08 viruses, Student’s *t*-test.

### NA enzyme kinetics

To investigate the effect of NA amino acid substitutions on NA enzymatic kinetics, we measured the *K*_m_ and *V*_max_ of NAs from recombinant influenza A and B viruses by using the fluorogenic MUNANA substrate (**Table 3**). The *K*_m_ of A/PR8 virus NAs carrying S331G and S331R NA substitutions was significantly increased relative to that of WT NA (*P* < 0.01), whereas the D342S and A395T NA substitutions decreased the *K*_m_ of recombinant B/YAM viruses (*P* < 0.01 and *P* < 0.05, respectively). The S331G and S331R NA substitutions increased the *V*_max_ of recombinant influenza A virus NAs (*P* < 0.001 and *P* < 0.01, respectively) (**Table 3**).

In contrast, the NAs of B/YAM viruses carrying D342S or A395T NA substitutions had a reduced *V*_max_ (*P* < 0.05). The higher *V*_max_ of the influenza A virus NA may facilitate the release and spread of A/PR8-S331G and A/PR8-S331R viruses, but the higher *K*_m_ indicates that the NA has a low affinity for its substrate. These results suggest that the NA substitutions contribute to the reduction in NA enzymatic activity.

### NA thermostability

**Fig 1:**
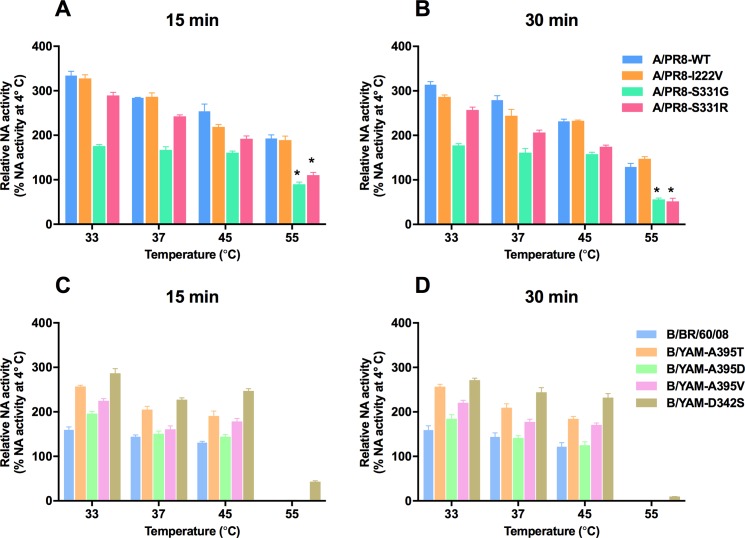
The NA thermostability of the recombinant influenza A and B viruses carrying a single NA substitution. Influenza A/PR8 (A, B) and B/YAM (C, D) viruses were incubated for 15 or 30 min at the indicated temperatures. Residual NA enzyme activity was assayed and is presented as a percentage of the NA activity at 4°C (unheated). The bars represent the mean of the relative NA activity ± SD for different temperatures from three experiments. *P < 0.05.

NA amino acid substitutions can contribute to NA protein stability [11]. Therefore, we determined the thermostability of mutant NAs by measuring the NA activity of the recombinant viruses after 15 and 30 min of incubation at different temperatures. For recombinant influenza A viruses, the NA activity of the A/PR8-I222V virus was stable at 33°C to 55°C, whereas the A/PR8-S331G and A/PR8-S331R viruses showed lower activity at 55°C (**Fig 1A and 1B**). The NA activity of recombinant B/YAM viruses carrying T/V/D changes at residue 395 and the B/BR/60/08 virus was undetectable after incubation at 55°C. In contrast, virus carrying the D342S NA substitution possessed high NA activity that was thermostable (**Fig 1C and 1D**).

### Replication kinetics

**Fig 2:**
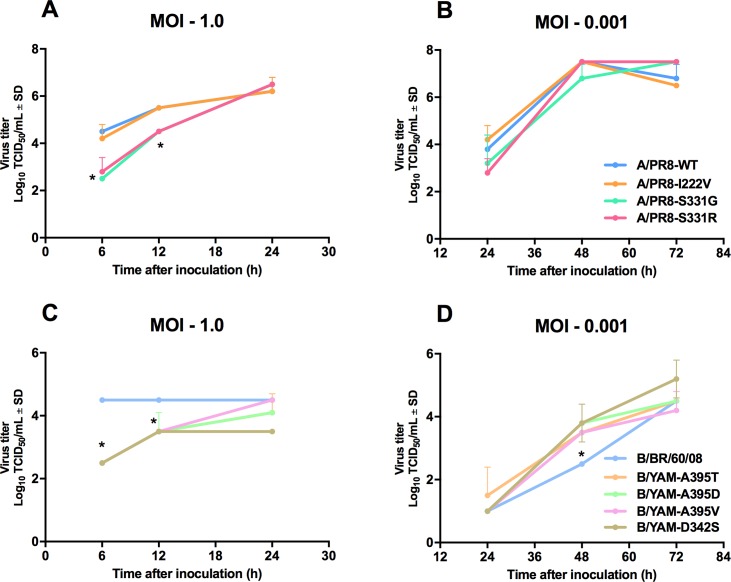
Replication kinetics in MDCK cells of recombinant influenza A and B viruses carrying a single NA amino acid substitution. MDCK cells were infected with recombinant influenza A and B viruses at an MOI of 1 (A, C) or 0.001 (B, D). Virus titers were determined by TCID_50_ assays in MDCK cells after incubation for 72 h at 35°C (for influenza A viruses) or 33°C (for influenza B viruses). Each data point represents the mean virus titer (log_10_TCID_50_/mL) ± the SD from three independent experiments. *P < 0.05.

To evaluate the fitness *in vitro* of recombinant influenza A and B viruses carrying NA amino acid substitutions, we studied their replication capacity in MDCK cells. At 6 and 12 h post-infection (hpi), the growth of A/PR8-I222V virus was similar to that of A/PR8-WT, but the A/PR8-S331G and A/PR8-S331R virus yields were significantly lower (*P* < 0.05) (**Fig 2A**). At 48 hpi, the yields of all recombinant influenza A viruses were comparable (range, 6.8–7.5 log_10_TCID_50_/mL) (**Fig 2B**). At 6 and 12 hpi, the recombinant influenza B virus yields were significantly lower than that of B/BR/60/08 virus (*P* < 0.05) (**Fig 2C**). At 48 hpi, all influenza B viruses replicated to significantly higher virus titers in MDCK cells when compared to B/BR/60/08 virus (*P* < 0.05) (**Fig 2D**). At 72 hpi, the viral titers of the recombinant influenza B viruses were 4.2 and 5.2 log_10_TCID_50_/mL. These results demonstrated that the NA substitutions S331G and S331R (on an A/PR8 background) and D342S, A395T, A395V, and A395D (on a B/YAM background) reduced the enzyme activity and thermostability of the NA. Although replication ability of recombinant influenza A and B viruses was heterogeneous at early time points, they showed at least equivalent yields as A/PR8-WT and B/BR/60/08 viruses at later time points. However, using B/BR/60/08 instead of the recombinant B/YAM virus with the wild-type NA is a caveat of this comparison.

### NA genetic stability

To determine whether NA substitutions introduced into the A/PR8 and B/YAM genetic backgrounds could be stably maintained *in vitro*, the NA genes were amplified from viruses before and after three passages in MDCK cells, and the NA sequences were determined. The I222V, S331G, and S331R NA substitutions were genetically stable in NA proteins on the genetic background of the A/PR8 virus. A diverse pattern was observed for influenza B viruses. The A395T and A395D substitutions were genetically stable and were maintained unchanged during three passages, whereas the D342S and A395V substitutions were unstable and the NAs reverted to WT after three passages (**Table 4**). These findings indicate that genetically unstable D342S and A395V NA substitutions are unlikely to emerge at a high frequency in influenza B viruses.

**Table 4:** Genetic stability of NA substitutions among recombinant influenza A and B viruses after three subsequent passages in MDCK cells.

Recombinant influenza virus	Passage in MDCK cells	
	0 passage	3^rd^ passage
*Influenza A viruses*		
A/PR8-I222V	V	V
A/PR8-S331G	G	G
A/PR8-S331R	R	R
*Influenza B viruses*		
B/YAM-D342S	S	D
B/YAM-A395T	T	T
B/YAM-A395D	D	D
B/YAM-A395V	V	A

## Discussion

In this study, we investigated the effect of novel NA amino acid substitutions on the NAI susceptibility, NA enzymatic properties, and replication capacity of influenza viruses *in vitro*. We generated recombinant (7:1) influenza A and B viruses containing the NA glycoprotein from viruses currently circulating in Thailand on the homogeneous genetic background of A/PR8 and B/YAM viruses for the recombinant influenza A and B viruses, respectively. The conserved I222 framework residue in the NA of influenza virus provides support to the catalytic residues [7]. In our study, the A/PR8-I222V virus was susceptible to NAIs in a phenotypic NA inhibition assay and possessed slightly increased NA enzymatic activity. It was previously reported that an influenza A(H3N2) virus with a single I222V NA amino acid substitution was susceptible to oseltamivir and possessed slightly higher NA enzymatic activity as compared to WT virus [28]. Our data revealed that recombinant A/PR8-I222V virus has only a minor effect on the NA enzymatic properties and virus replication efficiency in MDCK cells. However, the I222V NA substitution has a compensatory effect on the influenza A(H3N2) virus carrying E119V, and the presence of these two substitutions resulted in partially improved viral fitness in cell culture and resistance to oseltamivir [29].

Regarding the S331G/R, D342S, and A395T/D/V NA amino acid substitutions, these are found outside the NA active site, and viruses with these substitutions showed normal inhibition by NAIs. The S331G and S331R substitutions affected the kinetic properties of the NA enzyme, changing its binding affinity (*K*_m_) for MUNANA substrate. The *V*_max_ of the A/PR8-S331G and A/PR8-S331R virus NAs was nearly double that of the A/PR8-WT NA, demonstrating the compensatory effect as reflected in the lower replication capacity of viruses carrying S331G and S331R substitutions when compared to viruses with WT or I222V NAs. The significant decrease in NA activity and the increased *K*_m_ value demonstrated that S331G and S331R NA substitutions are deleterious to NA functions. The NA activity of the A/PR8-S331G and A/PR8-S331R viruses was decreased, and their viral titers in MDCK cells were lower than those of the WT virus at 6 and 12 hpi. The A/PR8-S331G and A/PR8-S331R viruses were characterized by a loss of NA activity at higher temperature (55°C), whereas PR8-WT and A/PR8-I222V viruses did not lose their NA activity. These data indicate that the viral replication capacity *in vitro* is dependent on the NA activity and the thermostability of the viruses [11, 30]. The B/YAM-D342S, B/YAM-A395T, and B/YAM-A395V viruses showed increased NA activity, whereas their *K*_m_ and *V*_max_ values decreased. These data suggest that there is an inverse relationship between NA activity and *K*_m_ and *V*_max_ values and that these amino acid substitutions are beneficial to NA functions. Moreover, B/YAM-D342S, B/YAM-A395T, and B/YAM-A395V viruses gave significantly higher viral yields at 48 hpi in MDCK cells, as compared to B/BR/60/08 virus. At higher temperature (55°C), the NA activity of recombinant influenza A viruses carrying NA substitutions was more stable than that of influenza B viruses. The A395V and D342S NA substitutions on the B/YAM background were unstable, and the NAs reverted to WT after three passages *in vitro*.

There were a few limitations in this study. As we could not generate a recombinant B/YAM virus with WT NA protein by using reverse genetics, we used the B/BR/60/08 virus, which is genetically similar to viruses circulating in Thailand, and compared the NA enzymatic properties and viral replication efficiency *in vitro*. Therefore, our results require validation using the B/YAM-WT NA virus. NAs from influenza A(H3N2) PCR-positive samples with I222V, S331G, and S331R NA substitutions were generated in the A/PR8 (H1N1) backbone. The replication efficiency of these viruses in MDCK cells may differ from that of natural isolates because of the effect of the HA-NA balance [31]. Although we used a validated and standardized method to determine influenza NA enzymatic properties using a whole virus preparation as a source of enzyme [27], this approach has limitations. Standardization of NA content based on TCID_50_ does not account for defective/non-infectious virions in the virus preparations, which may be enzymatically active. NA content can be reliably standardized in purified NA proteins; however, protein production on this scale, and when evaluating numerous viruses/proteins, is not readily achievable.

In conclusion, the recombinant influenza A and B viruses containing different NA substitutions located outside the NA active site exhibited normal inhibition by NAIs but differed in their NA enzymatic properties and their replication capacity *in vitro*. Continuous influenza antiviral surveillance is important for the identification of molecular markers of antiviral resistance and the characterization of NA enzyme functions and viral fitness.
